# Annotated Flickr dataset for identification of professional photographers

**DOI:** 10.1016/j.dib.2023.109511

**Published:** 2023-08-23

**Authors:** Rubén Gaspar Marco, Sofia Strukova, Félix Gómez Mármol, José A. Ruipérez-Valiente

**Affiliations:** Department of Information Engineering and Communications, University of Murcia, Murcia, Spain

**Keywords:** Image quality assessment, Photography capabilities, User expertise, Computational social science, Data mining, Social network

## Abstract

We collected and computed various data and statistics from a sample of Flickr users who uploaded photos to the platform in December 2021 and their photos, obtaining a final number of 27,516 users and 2,647,928 photos. Having the total number of photos uploaded and the number of photos uploaded in December by each user, we selected a representative sample of those whose activity was not overly concentrated in December and obtained data from those who specified their occupation. In addition to the data collected directly from Flickr, we enriched the dataset with new features resulting from the automated analysis of the photos and their comments. One of the most valuable features of this data collection is that each photo has three Image Quality Assessment scores representing aesthetic and technical aspects. For this, we used Convolutional Neural Networks trained with human-labeled data. Furthermore, we added labels to indicate whether the user is a professional photographer, so the data are specially prepared for supervised training.

Specifications Table

**Table:** 

Subject	Data Science
Specific subject area	Data-driven evaluation of capabilities
Data format	Analyzed, Filtered
Type of data	JSON
Data collection	We collected data from users who uploaded photos to Flickr in December 2021, specifically those who specified their occupation and were between the 5^th^ and 95th percentiles (total number of photos uploaded), such that they had not uploaded 20% or more of their total photos that month. We also collected and computed data from a random sample of 100 photos (or all, if less) from each.
Data source location	https://flickr.com/
Data accessibility	Repository name: Mendeley Data Data identification number: 10.17632/2nc8ytfw5x.1 Direct URL to data: http://dx.doi.org/10.17632/2nc8ytfw5x.1[1]

## 1 Value of the Data

• The dataset consists of a ready to use processed large-scale dataset from Flickr – an image hosting and video hosting service, that is also an online community.
• The features include aesthetic and technical rating of each photo calculated by Image Quality Assessment (IQA) popular neural networks.
• These data can be used by any researcher who wants to generate insights from a social network dedicated to photography.
• The data are already labeled on the photography professionalism of the users (a variable indicates for each user whether he/she is a self-proclaimed photographer by occupation) and therefore they are ready to perform supervised learning with ease.
• These data, methodologies and code sources are distributed under open license. In this sense, essential properties such as replicability, comparability and testability are ensured for each component.
• The code used for data extraction may be useful and can be further extended to obtain more data from Flickr.

## 2 Data Description

The dataset consists of two collections, namely, users’ features and photos’ features. Specifically, we have collected 2,647,928 photos uploaded by 27,516 unique users, with an average of 96 photos per user and 100 photos for more than 90% of users. These two collections are structured in JSON array of JSON objects format and are stored in the data files user_features.json and photo_features.json, respectively. Information about the features collected from users and the ones collected from photos is presented in Table 1 and Table 2, respectively.

**Table 1:** User's features*.

Name	Description	Domain
nsid	The anonymized identifier of the user.	UID
ispro	Boolean that indicates if the user has the paid membership Flickr Pro.	Boolean
photo_count	Total number of photos uploaded by the user to the platform.	N
join_date	User registration date.	Unix Time
occupation	Occupation indicated by the user.	Text
following	Identifiers of the users followed by the user.	JSON array of UID
following_n	Number of users followed by the user.	N
groups	Identifiers of the groups to which the user belongs.	JSON array of UID
groups_n	Number of groups to which the user belongs.	N
is_photographer	Boolean indicating whether the user is a photographer by profession, according to the occupation field. It was computed with the use of regular expressions in several languages that use the Latin alphabet. The regular expression includes the following terms – fot, phot, valokuv, zdjȩcie, dealbh, bild, grianghraf, nuotrauk, pictur, myndin, billed, ljosmyndari, ritratt*.	Boolean

⁎ Secondary data computed by researchers.

**Table 2:** Photo's features.

Name	Description	Domain
id	The anonymized identifier of the photo.	UID
owner	The anonymized identifier of the author of the photo. Foreign key referencing the nsid field of Table 1.	UID
views	The number of views the photo got.	N
dateuploaded	Date of the photo uploaded to Flickr.	Unix Time
lastupdate	Date of the last update of the photo meta- data (visits, favorites, comments, etc.).	Unix Time
tags	Tags attached to the photo.	JSON array of text
comments_n	Number of comments made on the photo page.	N
favorites	Identifiers of the users who added the photo to their list of favorite photos.	JSON array of UID
favorites_n	Number of users who added the photo to their list of favorite photos.	N
exif	Metadata of the photo in Exchangeable Image File Format	JSON object
groups	Groups in which the photo has been posted.	JSON array of UID
groups_n	Number of groups in which the photo has been posted.	N
width_o	Original width of the photo in pixels.	N
height_o	Original height of the photo in pixels.	N
width_downloaded	Width of the downloaded version of the photo in pixels.	N
height_downloaded	Height of the downloaded version of the photo in pixels.	N
kong_score	One of the aesthetic scores from Photo Aesthetics Ranking Network with Attributes and Content Adaptation [2] models implemented in [3]*.	[0*,* 1]
nima_score	The aesthetic score from NIMA [4] model implemented in [5]*.	[1*,*10]
nima_tech_score	The technical score from NIMA [4] model implemented in [5]*.	[1*,*10]
avg_subj	The average number of subjective words in posted comments computed with TextBlob Python library [6].	[0*,* 1]
avg_diff_words	The average number of difficult words in posted comments computed by Textstat Python library [7].	N
avg_read_time	The average reading time of posted comments computed by Textstat Python library [7].	N
avg_pola	The average polarity of posted comments computed by NLTK Python library [8].	[*−*1*,* 1]
avg_length	The average length of posted comments in characters.	N
avg_entropy	The average entropy of posted comments.	N

⁎ Secondary data computed by researchers.

**Fig. 1:**
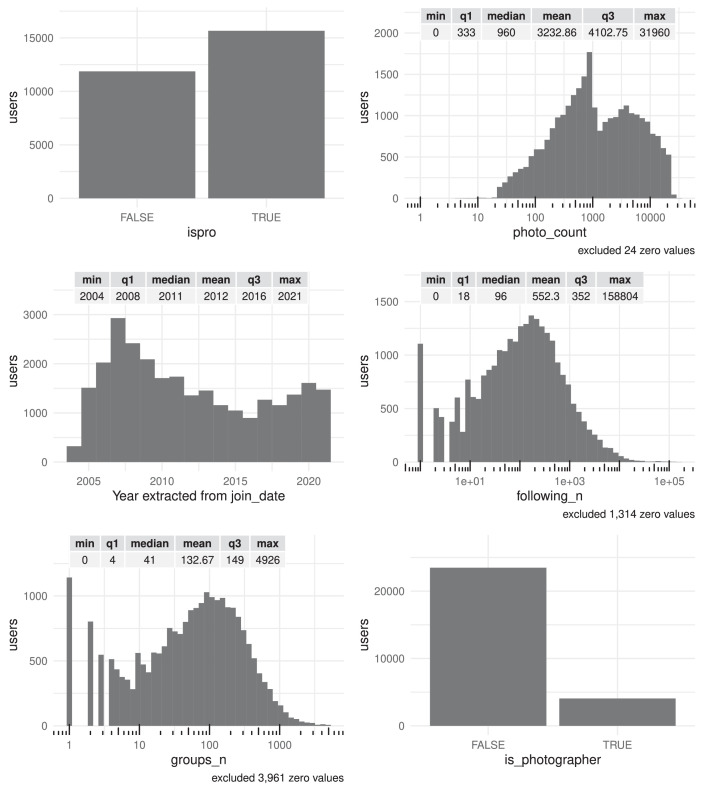
Summary statistics (distribution, minimum, maximum, quartiles and mean) of users' features ispro, photo_count, join_date, following_n, groups_n and is_photographer. There are tick marks with diminishing spacing on those axes that have been log-scaled for readability.

**Fig. 2:**
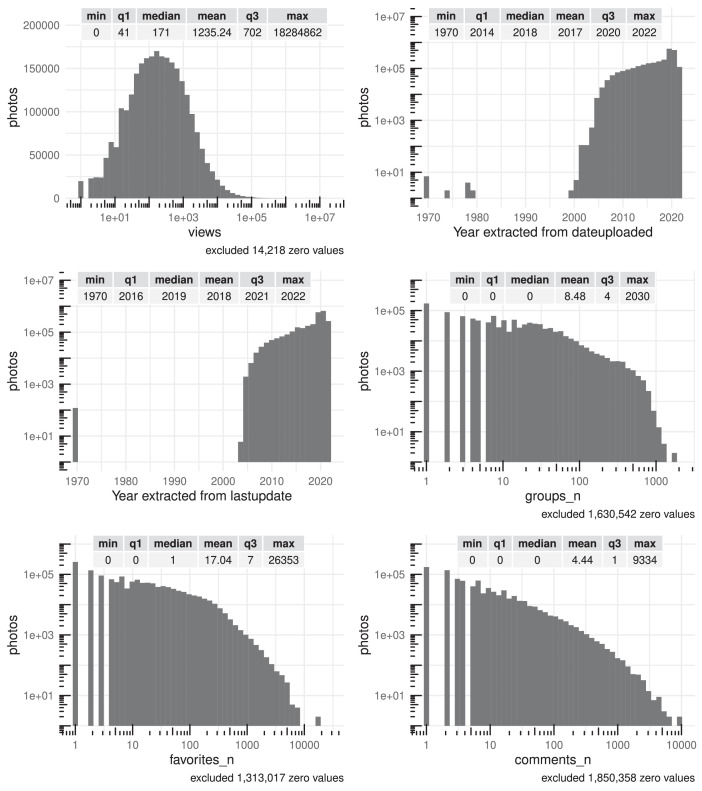
Summary statistics (distribution, minimum, maximum, quartiles and mean) of photos' basic features views, dateuploaded, lastupdate, groups_n, favorites_n and comments_n. There are tick marks with diminishing spacing on those axes that have been log-scaled for readability.

**Fig. 3:**
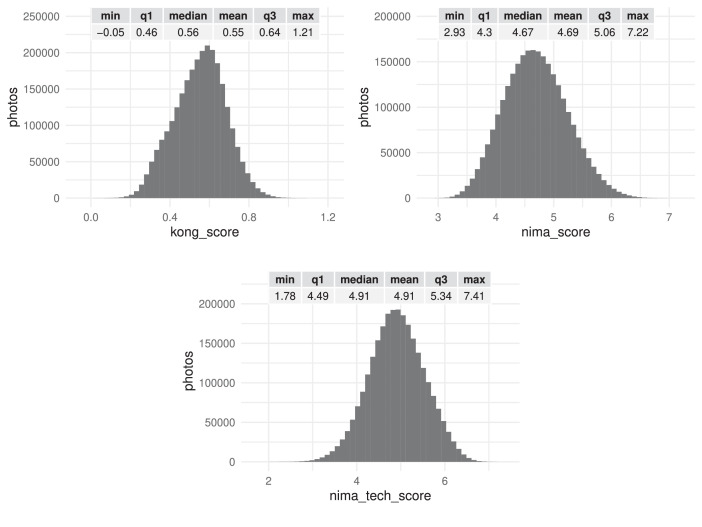
Summary statistics (distribution, minimum, maximum, quartiles and mean) of IQA scores kong_score, nima_score and nima_tech_score. There are tick marks with diminishing spacing on those axes that have been log-scaled for readability.

**Fig. 4:**
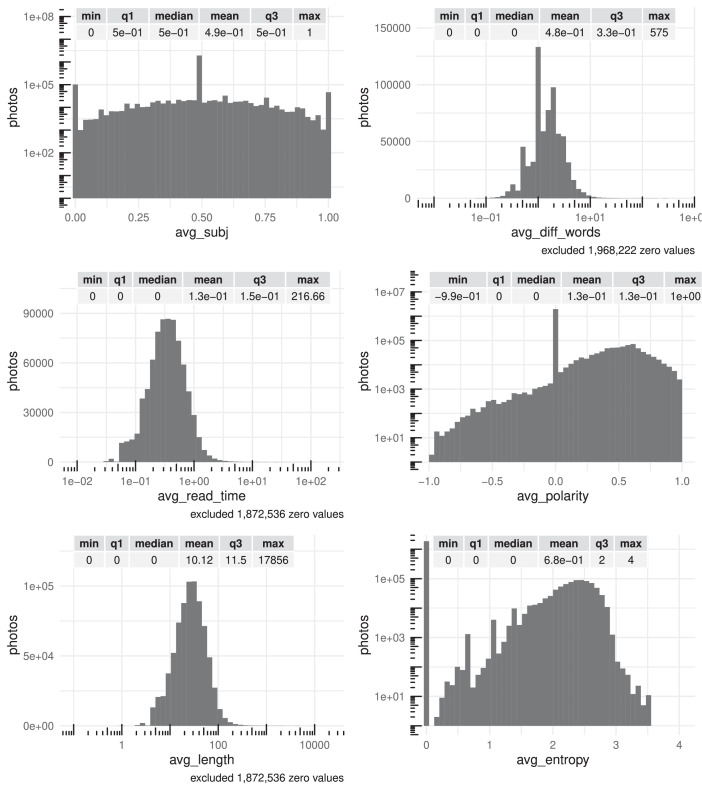
Summary statistics (distribution, minimum, maximum, quartiles and mean) of photos' computed features about comments avg_subj, avg_diff_words, avg_read_time, avg_polarity, avg_length and avg_entropy. There are tick marks with diminishing spacing on those axes that have been log-scaled for readability.

Fig. 1, Fig. 2, Fig. 3, Fig. 4 show summary graphs of the distribution of the variables collected. We have collected a reasonably heterogeneous sample of users, which is a good indicator that the sample is representative. It is worth noting that in the variable photo_count the median is expected to be very close to 1,000, as this is the maximum number of photos that can be uploaded with a free account (without a Flickr Pro subscription). Also, the number of Pro users is reasonably balanced in the dataset. We also noticed that several Flickr date records, specifically the upload and last update dates, are corrupted. These include dates prior to the creation of the platform (2004). In fact, several of these values correspond to the Unix epoch, the minimum date representable by Unix time.

## 3 Experimental Design, Materials and Methods

**Fig. 5:**
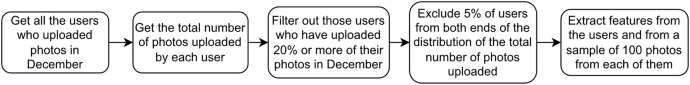
Methodology diagram.

The methodology followed for data collection is summarized in Fig. 5 and will be explained in detail in the following sections.

### 3.1 User selection

To obtain a representative and comprehensive sample of the platform's active users, we intended (see LIMITATIONS) to take those users whose profiles showed activity (in the sense of the number of uploaded photos) during December 2021.

The strategy followed to do so was to search for all the photos uploaded in December. Among the optional parameters of the search, apart from the upload date range mentioned next, we set content_type to 1 (only photos, discarding screenshots and ‘other’) and media to ‘photos’ (discarding videos).

We split the search into multiple requests, each one with the minimum upload date range, in order to minimize the number of search results. This is necessary for the result to be as comprehensive as possible (see LIMITATIONS). After each request, we took the maximum value of the upload date of the results so that the next request is for photos uploaded within 5 minutes of the last photo in the result. This ensures that the next search request does not intersect with the previous one, as Flickr does not always respect the upper time limit requested. At the end of this process, we had 225,590 users who had uploaded photos in December.

The next step was to collect activity data from the harvested users to identify the platform's regular users. During the search for photos uploaded in December, we counted the number of photos uploaded that month by each user collected. Afterward, we determined the total number of photos uploaded by each harvested user. This value can be extracted directly from the information provided by the API about a particular user. However, since an API request had to be made for each user, this step took about 80 hours.

Utilizing the two variables - the aggregate number of photos uploaded and the quantity of photos uploaded in December, we filtered out those users whose activity (number of photos uploaded) in December was equal to or greater than 20% of their total activity in order to filter out those users without a minimum activity on the platform. Our analysis also identified anomalous users at both ends of the distribution of total uploaded photos. To mitigate the impact of such outliers, we also filtered out the 5% of users from both ends of the distribution of total photos uploaded to avoid outliers; that is, we kept the range between the 5th and 95th percentiles. At the end of this stage, we had a total of 151,468 users.

Since we were interested in labeling the dataset according to whether the user was a professional photographer or not, we ignored all users whose occupation field was empty, to avoid bias derived from assuming their profession. This was checked on the run while iterating over all users. Excluding these and those users who had deleted the account between the previous stage and the current one, we finally obtained 27,516 users.

### 3.2 Photo sampling

Despite our attempts to reduce the latency in data extraction, the time cost required to take into account all the users’ photos was too high. For this reason, we decided not to process more than 100 photos per user, which would already take about two months. Therefore, for each user, we took all his photos if he had less than 100 and randomly sampled 100 otherwise.

### 3.3 Feature extraction

For the entire data collection process, we prepared a script that makes extensive use of [9]. This script is available on GitHub [10]. We will detail below some of the most important aspects of it.

### 3.4 Multithreading

**Fig. 6:**
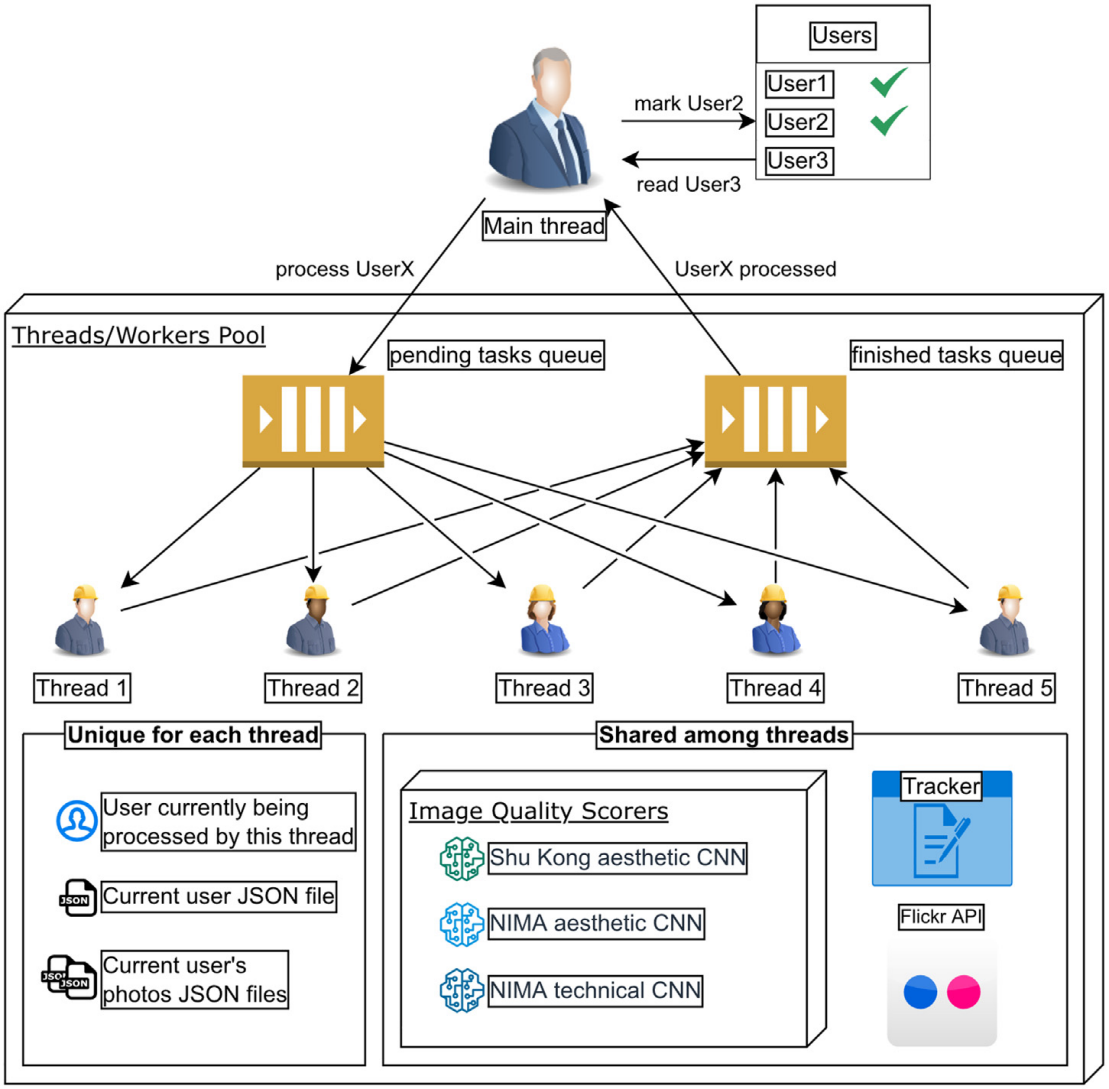
Multithreading diagram.

**Fig. 7:**
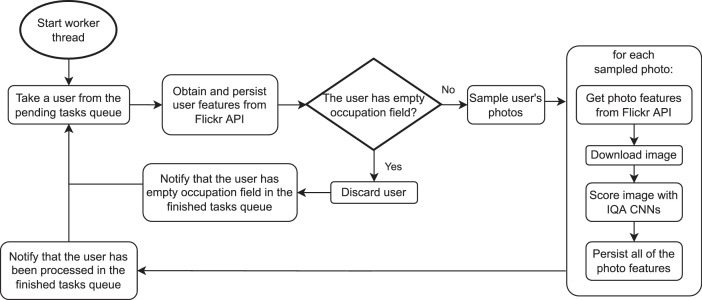
Worker threads flow.

The data extraction has been implemented using multithreading to avoid the interruption of the whole pipeline when waiting for the response from the Flickr API. For each user, the main thread enters the order to extract the user's features in the queue of pending tasks, from where one of the worker threads takes the order and is exclusively in charge of processing that user and his photos. The worker threads make shared use (with the help of locks) of the Flickr API, IQA neural networks, and a Tracker object that allows keeping track of the number of queries performed, the number of photos processed, and other statistics. Once the worker is done with the user, it notifies it through the completed task queue, where the main thread can pick it up and mark that user accordingly. Fig. 6, Fig. 7 represent the resulting schema.

The main thread keeps, using the finished tasks queue, an updated list of users that have been completely processed. For those that have not, it also notes the corresponding reason: empty occupation field, unhandled exception, deletion from Flickr, or have not been processed yet. This allows that, in case of unexpected program termination, it can return to a state very close (what has not yet been written to disk is not recoverable) to the one it was in before. If the user is half processed, we check what data is on disk and follow the usual process with the not-yet processed data.

Other measures that make the program robust are insistent API calls, which are repeated in case of connection error, quite frequent during data extraction, primarily due to “Server Error 5xx” HTTP response status codes.

### 3.5 Image quality assessment models

Aesthetic Visual Analysis (AVA) [11] is a dataset for IQA that contains over 250,000 images along with a rich variety of metadata including a large number of aesthetic scores for each image. Each AVA photo is scored by an average of 200 people in response to photography contests. To score the quality of Flickr users’ photos, we considered to use deep learning models that are a powerful tool for extracting insights from big data [12]. Therefore, we searched for the most popular models that had been tested on this dataset and were publicly accessible (see LIMITATIONS).

The feature kong_score comes from one of the models of the work done by Shu Kong et al. in [2], in particular, from the model used in the demo of their official repository [3]. They modified the AlexNet Convolutional neural network (CNN) [13] by adding layers for regression and branching (one base regression branch and 11 attribute branches) to the network and including additional activation layers that are trained to encode informative attributes (color harmony, vivid color, etc.). These branches are rejoined in two final layers for the final regression of the quality score.

The features nima_score and nima_tech_score come from the model implemented by Idealo [5] based on [4]. As baseline architecture, the authors use the pre-trained CNN MobileNet (they use the weights as initialization, but afterward all the weights were trained) on the ImageNet dataset [14]. In addition, they replace the last layer of the baseline CNN with a fully-connected layer with 10 neurons followed by soft-max activations. With the same architecture, they train two different models, the aesthetic one with AVA and the technical one with the Tampere Image Database [15], leading to the state-of-the-art performance for both tasks. The model predicts the distribution of the ratings that a photo would get by a group of humans, but we use in both cases (aesthetic and technical) the mean as the rating of the photo quality.

### 3.6 Memory saving

The NIMA model takes images of 224 × 224 pixels size as input and Photo Aesthetics Ranking Network with Attributes and Content Adaptation model works with 227 × 227 pixels images. For this reason, obtaining images larger than this size has no benefit, with the disadvantage that they take up more memory and time. Thus, among the available sizes, we choose the smallest one that provides an image whose smallest side is at least 230 pixels. We use this margin of 3 pixels because the API occasionally returns images with fewer pixels than what it says.

**Fig. 8:**
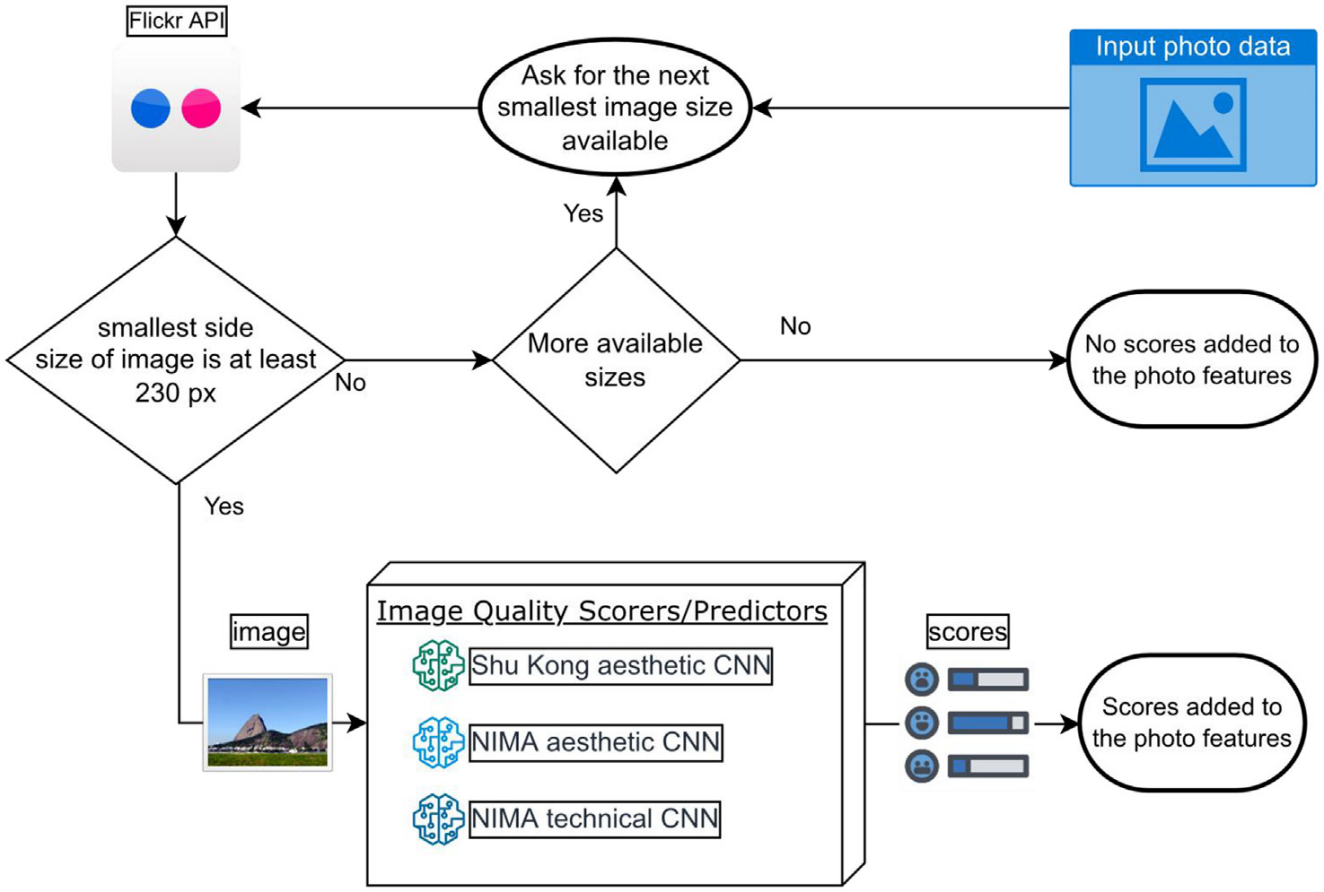
Photo scoring process.

From the images uploaded by users, we are only interested in the scores provided by the IQA models. Furthermore, we could not make the images public without the consent of the users and without filtering by the photo's license or by whether people appear in the image. Thus, the images are downloaded and decompressed into RAM and fed in PIL format to the IQA models, which as part of their preprocessing, must convert them to the format taken by the input layer of their CNN (see Fig. 8). After the images have been scored, they are released from memory.

### 3.7 Comments preprocessing

Numerous data preprocessing phases were necessitated by the review of the comments. First, we changed all the comments to lowercase we converted every comment to lowercase letters. Next, we utilized Python Demoji library [16] in order to replace emojis in comments with their description codes. Moreover, we cleaned the comments from hyperlinks and non-alphanumeric text. Finally, we removed empty comments (including those that contained solely stop words).

## 4 Limitations

During data extraction, we encountered several limitations, mainly due to the Flickr API undefined behaviors. The most relevant problems are listed below:

1. Although we had multiple options for IQA models, we ended up using only two, as it was not possible to find accessible versions of the others.
2. The authors of Photo Aesthetics Ranking Network with Attributes and Content Adaptation did not release all the models used in their paper [9], so we had to settle for the model used in the demo in the official GitHub repository of the article [3].
3. Much of the relevant information about the behavior of the API methods and the format of its responses is undocumented or scattered in developer blog follows: https://code.flickr.net/.
4. The Flickr API search method has undefined behaviors when the total amount of results is greater than 4,000. These behaviors include empty or repeated results pages or changeable number of results.
  a. The number of results of a search whose only parameter is the upload date range exceeded this amount, even with the smallest possible range. For this reason, we cannot ensure the completeness of the data.
5. We have not found documentation on what is the minimum possible time range in which the API allows to search for photos (respecting the range), but when the specified time range is less than 10 minutes long, the API usually returns photos outside the range, although seemingly respecting the lower limit.
6. Despite specifying restriction parameters in the photo search (content_type to discard screenshots and ‘other’ and media to discard videos), we have found that several of the photos are illustrations or 3D modeling scenes.

## Ethics Statement

The data distributed with this article are intended for non-commercial research. The published user data have been fully anonymized by hashing all identifiers (users, photos, and groups) and omitting sensitive data such as the users’ images or profile descriptions. In addition, we have complied with all Flickr Policies & Guidelines, available at https://www.flickr.com/help/terms.

## CRediT authorship contribution statement

**Rubén Gaspar Marco:** Conceptualization, Methodology, Software, Formal analysis, Data curation, Writing – original draft, Visualization. **Sofia Strukova:** Conceptualization, Methodology, Software, Formal analysis, Writing – review & editing. **Félix Gómez Mármol:** Conceptualization, Writing – review & editing, Supervision, Project administration. **José A. Ruipérez-Valiente:** Conceptualization, Writing – review & editing, Supervision, Project administration.

## Data Availability

Annotated Flickr dataset for identification of professional photographers (Original data) (Mendeley Data).
